# Thermally Activated Delayed Fluorescent Gain Materials: Harvesting Triplet Excitons for Lasing

**DOI:** 10.1002/advs.202200525

**Published:** 2022-03-28

**Authors:** Chang‐Cun Yan, Xue‐Dong Wang, Liang‐Sheng Liao

**Affiliations:** ^1^ Institute of Functional Nano & Soft Materials (FUNSOM) Jiangsu Key Laboratory for Carbon‐Based Functional Materials & Devices Soochow University 199 Ren'ai Road Suzhou Jiangsu 215123 P. R. China; ^2^ Macao Institute of Materials Science and Engineering Macau University of Science and Technology Taipa Macau SAR 999078 P. R. China

**Keywords:** electrically pumping, gain material, organic solid‐state laser, reverse intersystem crossing, thermally activated delayed fluorescent

## Abstract

Thermally activated delayed fluorescent (TADF) materials have attracted increasing attention because of their ability to harvest triplet excitons via a reverse intersystem crossing process. TADF gain materials that can recycle triplet excitons for stimulated emission are considered for solving the triplet accumulation problem in electrically pumped organic solid‐state lasers (OSSLs). In this mini review, recent progress in TADF gain materials is summarized, and design principles are extracted from existing reports. The construction methods of resonators based on TADF gain materials are also introduced, and the challenges and perspectives for the future development of TADF gain materials are presented. It is hoped that this review will aid the advances in TADF gain materials and thus promote the development of electrically pumped OSSLs.

## Graphical abstract

Thermally activated delayed fluorescent (TADF) gain materials with the ability to harvest triplet excitons for stimulated emission are predicted to resolve the triplet accumulation problem in electrically pumped organic solid‐state lasers. However, TADF gain materials are still in their early stage of development. Herein, the pioneering work is summarized, and the challenges are discussed.

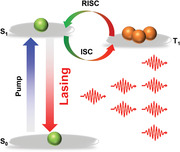

## 1 Introduction

Laser technology has been one of the most important inventions in the last century and has been widely applied in various fields, such as telecommunications, medical diagnosis, industrial manufacturing and academic research. With the first discovery of ruby lasers in the 1960s,^[^ ^1^ ^]^ organic materials have been considered to be used as gain materials to achieve lasing owing to their advantages of a large excited cross‐section, tunable emission wavelength and low‐cost manufacturing process.^[^ ^2^, ^3^ ^]^ In 1966, Sorokin and Lankard reported the first dye laser.^[^ ^4^ ^]^ Since then, organic lasers have undergone rapid development. In 1967, Soffer and McFarland doped rhodamine 6G in polymethylmethacrylate as an organic gain medium, which led to the development of the first organic solid‐state laser (OSSL).^[^ ^5^ ^]^ Compared with liquid dye lasers, OSSLs are more attractive because of their advantages of stable operation, low weight, and easy miniaturization and integration.^[^ ^6^ ^]^ The innovation of gain materials plays an important role in the development of OSSLs.^[^ ^7^ ^]^ Organic semiconducting materials exhibit excellent luminescence properties and carrier mobility. Owing to their excellent photoelectric properties, organic semiconductor materials have been widely used in organic optoelectronic devices, such as organic light‐emitting diodes (OLEDs),^[^ ^8^, ^9^, ^10^, ^11^ ^]^ organic solar cells,^[^ ^12^, ^13^, ^14^ ^]^ and organic thin‐film transistors.^[^ ^15^, ^16^ ^]^ In 1996, organic conjugated polymers poly(p‐phenylenevinylene) (PPV) and PPV derivatives with semiconductor properties were first used as gain media by Heeger and Friend (and their co‐workers) respectively.^[^ ^17^, ^18^ ^]^ Since then, organic semiconductor materials have received extensive attention as gain media.^[^ ^19^, ^20^ ^]^ The successful application of organic semiconductor materials in OSSLs facilitates combining OSSLs with electricity.

For organic gain materials, the stimulated emissions were mainly achieved based on a population inversion mechanism.^[^ ^20^ ^]^ In contrast to inorganic materials, organic molecules have several vibrational energy levels in both the ground and excited states. An inherent four‐level energy system can be formed by these vibrational energy levels, which guarantees an effective population inversion and facilitates stimulated emission.^[^ ^21^ ^]^ For some organic molecules, phototautomerization reactions, such as excited‐state intramolecular proton transfer (ESIPT), can occur during excitation, in which more effective four‐level energy systems can be formed by the ground and first excited states of normal forms and tautomer forms.^[^ ^22^, ^23^, ^24^, ^25^ ^]^ This type of more effective four‐level energy system can lead to easier population inversion and further result in a lower laser threshold. To date, various organic materials, such as dyes, polymers, metal complexes, and semiconductive molecules, have been developed and investigated as gain materials in OSSLs. However, most of these materials exhibit high optical gain properties only under photopumped conditions. Electrically pumped OSSLs remain a challenge.^[^ ^26^, ^27^ ^]^

**Figure 1:**
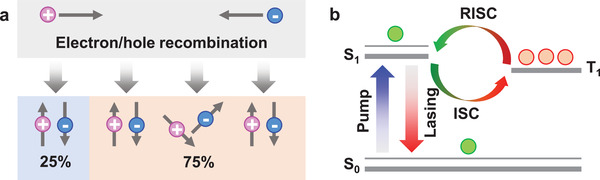
a) Schematic of the electron–hole combination in organic electroluminescent devices. b) Proposed energy diagram of harvesting triplet excitons for lasing via the RISC process for TADF gain materials.

It is well known that 25% of singlet excitons and 75% of triplet excitons are generated during the recombination of electrons and holes under current‐injection conditions in organic electroluminescent devices according to spin quantum statistics (**Figure** **1**a).^[^ ^28^ ^]^ Phosphorescent emission from triplet excitons is usually in relatively low efficiency owning to the spin‐forbidden nature, which makes it difficult to achieve optical gain. Although a recent example of light amplification by stimulated emission in phosphorescent materials has been reported, in most cases, only singlet excitons can be used in the stimulated emission process of organic materials.^[^ ^29^ ^]^ Therefore, under current‐injection conditions, the lasing threshold would be three times higher than it should be, even though it is already predicted to be very high.^[^ ^30^ ^]^ In addition, as the lifetime of the first triplet state (T_1_) is usually much longer (over three orders of magnitude) than that of the first singlet state (S_1_), serious triplet accumulation occurs under a high current density. Triplet accumulation can lead to triplet–triplet annihilation and triplet–singlet annihilation through T_1_ absorption, which reduces the S_1_ population and aggravates optical losses in the process of light amplification.^[^ ^31^ ^]^ Therefore, minimizing the optical losses caused by T_1_ excitons is an important problem for the realization of electrically pumped OSSLs. To achieve this, strategies for managing T_1_ excitons have been employed. For example, T_1_ excitons can be eliminated by introducing triplet annihilators such as oxygen,^[^ ^32^ ^]^ cyclooctatetraene,^[^ ^32^, ^33^, ^34^ ^]^ and anthracene derivatives.^[^ ^34^, ^35^ ^]^ Thus, most excitons are destroyed, and the stability of the laser devices is significantly reduced by the active components or heat generated by triplet annihilation.^[^ ^32^, ^36^ ^]^

Thermally activated delayed fluorescent (TADF) materials can transfer T_1_ excitons back to S_1_ excitons through a reverse intersystem crossing (RISC) process with the aid of thermal activation.^[^ ^9^, ^10^, ^37^ ^]^ The proposed energy diagram of the TADF gain materials under current injection conditions is shown in Figure 1b. Both S_1_ and T_1_ excitons can be generated under current injection conditions. The S_1_ excitons can return to the ground state (S_0_) by fluorescence emission or stimulated emission. For T_1_ excitons, the direct S_0_ ← T_1_ transition is forbidden. As the singlet–triplet energy gap (Δ*E*_ST_) is very small for TADF materials, long‐lifetime T_1_ excitons are converted into S_1_ excitons via RISC with the aid of thermal activation. The regenerated S_1_ excitons can then return to the S_0_ state by fluorescence emission or stimulated emission, which is known as delayed fluorescence or lasing. Thus, TADF materials can effectively utilize triplet excitons for fluorescence or lasing. In theory, 100% of the excitons produced by electrical excitation in TADF materials can be used. Therefore, in TADF OLEDs, an internal quantum efficiency close to 100% and an external quantum efficiency (EQE) over 25% have been achieved, which exceed the efficiency limitation.^[^ ^38^, ^39^ ^]^ Although the realization of electrically pumped OSSLs is still difficult, photopumped continuous‐wave or quasi‐continuous‐wave lasing, which also faces triplet accumulation problems, has already been achieved by the reuse of T_1_ excitons.^[^ ^40^, ^41^, ^42^ ^]^ Based on these previous studies, TADF molecules with laser activity have become the most likely organic gain material for electropumped organic lasers. Further progress has been made in recent years.^[^ ^43^, ^44^, ^45^, ^46^, ^47^, ^48^, ^49^, ^50^, ^51^, ^52^ ^]^

Since the first attempt to use TADF materials in OLEDs,^[^ ^53^, ^54^ ^]^ various TADF materials have been designed and synthesized.^[^ ^55^, ^56^, ^57^ ^]^ However, only some of these materials have been reported to exhibit laser activity.^[^ ^43^, ^44^, ^45^, ^46^, ^47^, ^48^, ^49^, ^50^, ^51^, ^52^ ^]^ The relationship between the molecular structures of TADF materials and their laser properties remains unclear. Recent progress in TADF gain materials is reviewed in this paper to illustrate the recent advances in TADF gain materials, raise awareness of this new field and extract the fundamental design principles and strategies of TADF gain materials. First, the fundamental photoelectric properties and laser performances of the reported TADF materials are summarized, based on which the design principles of TADF gain materials are proposed. Subsequently, methods for achieving lasing based on TADF materials are introduced. Finally, the challenges and perspectives for developing novel TADF gain materials are presented.

## 2 TADF Gain Materials

**Figure 2:**
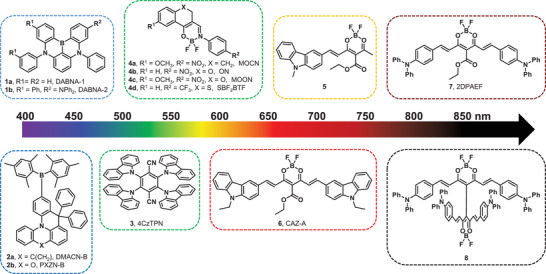
Chemical structures of reported TADF gain materials.

In recent years, TADF gain materials have drawn increasing attention owing to their potential applications in electrically pumped OSSLs. Several pioneering studies have been conducted to date.^[^ ^43^, ^44^, ^45^, ^46^, ^47^, ^48^, ^49^, ^50^, ^51^, ^52^ ^]^ The molecular structures of the reported TADF gain materials are summarized in **Figure** **2** and sorted by laser wavelength from blue to near‐infrared. The photoelectric properties and laser performance under optically pumped conditions are summarized in **Table** **1**. As previously reported, for classical TADF molecules, large steric electron donor and electron acceptor units were used to induce the spatial separation of the highest occupied and lowest unoccupied molecular orbitals (HOMO and LUMO, respectively) to reduce Δ*E*_ST_.^[^ ^10^, ^55^, ^56^ ^]^ Because of their strong electron‐donating ability, nitrogen‐containing aromatic groups, such as carbazole, diphenyl amine, phenoxazine, and their derivatives, are often used as donors. Nevertheless, several types of acceptor groups can be used to tune the photoelectric performance of TADF materials. These rules can also be applied to TADF gain materials. In this section, representative materials based on acceptor type are discussed in detail.

**Table 1:** Photophysical properties and laser performances of reported TADF materials

Comp.	*λ*_FL_ [nm]^a)^	*τ*_FL_ [ns]^b)^	*τ*_TADF_ [µs]^c)^	PLQY^d)^	Δ*E*_ST_ [eV]^e)^	Amplification process^f)^	Sample state	*λ*_ASE or laser_ [nm]	P_th_ [µJ cm^−2^] (at pulse length)^g)^	Ref.
**1a**	460^h)^	8.8^h)^	93.7^h)^	0.8^h)^	0.18	ASE	6 wt% in *m*CBP film	460	2.8 (0.8 ns)	[43]
	–	–	–	–	–	Lasing	Doped in *m*CBP microplate	480	5.5 (100 fs)	[52]
**1b**	469^h)^	6.0^h)^	65.3^h)^	0.90^h)^	0.14	ASE	6 wt% in *m*CBP film	494	1.6 ± 0.3 (0.8 ns)	[43]
**2a**	454^i)^	4.0^j)^	0.8^j)^	0.93^j)^	0.28	ASE	10 wt% in *m*CBP film	468	3.3 (0.8 ns)	[44]
**2b**	430^i)^	5.4^j)^	0.3^j)^	0.90^j)^	0.27	ASE	10 wt% in *m*CBP film	448	12.0 (0.8 ns)	[44]
**3**	520^k)^	10.7^k)^	1.4^k)^	0.71^k)^	0.06	Lasing	3 wt% in PS microsphere	563	84–95 (200 fs), 88 (450 fs)	[45]
	560^l)^	11.2^l)^	1.8^l)^	0.43^l)^	0.02	Lasing	2.7 wt% in *m*CBP microplate	567	30.5 (100 fs)	[52]
**4a**	502^m)^ (539)^n)^	10.5^m)^	3.5^m)^	0.65^m)^	0.19 (S_1_–T_2_)	Lasing	Microcrystal	525	3.5 (150 fs)	[46]
**4b**	532^m)^ (561)^n)^	9.7^m)^	2.0^m)^	0.80^m)^	0.02 (S_1_–T_2_)	Lasing	Microcrystal	561	5.0 (150 fs)	[46]
**4c**	580^m)^ (588)^n)^	5.5^m)^	9.9^m)^	0.70^m)^	0.12 (S_1_–T_2_)	Lasing	Microcrystal	650	3.0 (150 fs)	[46]
**4d**	560^m)^ (556)^n)^	0.8^m)^ (0.4)^n)^	0.9^m)^ (0.3)^n)^	0.12^m)^	0.12 (S_1_–T_2_)	Lasing	Microcrystal	560	10.6 (150 fs)	[47]
**5**	610^n)^	2.3^n)^	3.8^n)^	0.17^n)^	0.24	ASE	Microcrystal	610	23.6 (150 fs)	[51]
**6**	653^o)^	1.3^o)^	191^o)^	0.13^o)^	0.26	Lasing	4 wt% in *m*CBP microring	677–700	4.0 (150 fs)	[50]
**7**	706–782^p)^	0.5, 2.3^q)^	183, 28^q)^	0.70 (721)^q)^	0.37	ASE	Doped in *m*CBP film^p)^	738–798^p)^	4.7–36.7 (0.8 ns)^p)^	[48]
	–	–	–	–	–	Lasing	Doped in *m*CBP microplate	740	3.6 (100 fs)	[52]
**8**	760–801^r)^	–	–	0.45^s)^	0.30	ASE	Doped in *m*CBP film^r)^	801–860^r)^	7.5–91.3 (0.8 ns)^r)^	[49]

^a)^ Photoluminescence emission maxima;

^b)^ Lifetime of prompt fluorescence;

^c)^ Lifetime of delayed fluorescence;

^d)^ Photoluminescence quantum yield;

^e)^ Singlet–triplet energy gap;

^f)^ ASE: amplified spontaneous emission;

^g)^ Threshold;

^h)^ Measured in doped film (1 wt% in 3,3’‐bis(*N*‐carbazolyl)‐1,1’‐biphenyl (*m*CBP));

^i)^ Measured in solution (toluene);

^j)^ Measured in doped film (20 wt% doped in bis[2‐(diphenylphosphino)phenyl]ether oxide);

^k)^ Measured in doped film (3 wt% doped in polystyrene (PS));

^l)^ Measured in doped microcrystal (2.7 wt% doped in *m*CBP);

^m)^ Measured in solution (dichloromethane);

^n)^ Measured in crystal state;

^o)^ Measured in doped microcrystal (4 wt% doped in *m*CBP);

^p)^ Measured in doped film (2–60 wt% in *m*CBP);

^q)^ Measured in doped film (6 wt% in *m*CBP);

^r)^ Measured in doped film (2–40 wt% in *m*CBP);

^s)^ Measured in doped film (2 wt% in *m*CBP).

### 2.1 TADF Gain Materials with Triarylboron‐Type Acceptors

Owing to the vacant p‐orbital in the central boron atom, the triarylboron groups exhibit strong electron‐attracting properties. Donor‐acceptor (D‐A)‐type molecules containing amine‐based donors and triaryboron acceptors often exhibit strong intramolecular charge transfer properties. In addition, nitrogen and boron atoms have opposite resonance effects, which can facilitate the efficient separation of HOMOs and LUMOs.^[^ ^58^ ^]^ In 2017, Adachi et al. reported two triphenylboron‐based materials (compounds **1a** and **1b**), in which two nitrogen atoms joined with adjacent phenyl groups to form a rigid polycyclic aromatic framework.^[^ ^43^ ^]^ For both compounds, Δ*E*_ST_s are very small (0.18 and 0.14 eV, respectively), which ensured their TADF activities. In addition, both compounds **1a** and **1b** showed high oscillator strengths of the S_0_–S_1_ transition (*f* = 0.205 and 0.415, respectively), which are beneficial for obtaining large excited cross‐sections and thus beneficial for realizing stimulated emission. For compound **1b**, the introduction of substituents can improve the oscillator strength without affecting the localization of the molecular orbitals. Accordingly, a lower amplified spontaneous emission (ASE) threshold was achieved for compound **1b** than for compound **1a** under excitation by a pulsed laser. However, because of the low rate of RISC (*k*_RISC_), which is more than four orders of magnitude lower than the radiative decay rate (*k*_r_) of S_1_, the contribution of RISC to the light amplification process is negligible. Nevertheless, this study demonstrates that TADF materials can be used as gain media for OSSLs.

More recently, based on previously reported works, Liao et al. have developed compounds **2a** and **2b**.^[^ ^44^ ^]^ As shown in Figure 2, a mesitylboron group was used as the acceptor and phenoxazine and acridine derivatives were used as donors. Bulky mesityl groups were introduced to the acceptor segment to improve the stability of the aromatic boron group. For the donor segments, a single intramolecular lock was introduced to increase molecular rigidity and flatness. Both compounds exhibited TADF activities and ASE characteristics with low thresholds. Theoretical calculations showed that the S_1_ states of locked structures have higher oscillator strengths (*f* = 0.37 and 0.32 for compounds **2a** and **2b**, respectively) and more locally excited character compared with the lock‐free counterparts without ASE activities. As evident from the above study, increasing the molecular rigidity contributes to the improvement of the oscillator strength of the S_0_–S_1_ transition, thus leading to easier lasing.

### 2.2 TADF Gain Materials with Cyano‐Based Acceptors

The cyano group has a strong electron‐withdrawing ability. 2,3,5,6‐Tetrakis(carbazol‐9‐yl)‐1,4‐dicyanobenzene (4CzTPN, compound **3**), with two cyano groups and four carbazole groups connected to a benzene skeleton, is a classical TADF molecule.^[^ ^9^ ^]^ The use of 4CzTPN in OLEDs to achieve a high EQE has been reported for ten years.^[^ ^9^ ^]^ Owing to its high photoluminescence quantum yield and short delayed fluorescence lifetime, 4CzTPN was chosen as the gain material by Zhao et al. to develop an OSSL.^[^ ^45^ ^]^ When 4CzTPN was doped in polystyrene (PS) microspheres, lasing was achieved under photopumped conditions, even though the threshold was slightly high. Notably, the lasing threshold can be reduced by increasing the pump laser frequency. This indicated that T_1_ excitons can be effectively used in the RISC process. Therefore, a high *k*_RISC_ is a prerequisite for the efficient utilization of triplet excitons to achieve lasing for TADF materials.

### 2.3 TADF Gain Materials with Difluoroboron‐Based Acceptors

Difluoroboron complexes such as acetylacetonate boron difluoride can be used as acceptor units in TADF materials because of their electron‐deficient properties. In 2018, a boron difluoride curcuminoid complex (compound **7** in Figure 2) with two triphenylamine groups as donors and an acetylacetonate boron difluoride derivative as an acceptor was designed to achieve near‐infrared emission.^[^ ^48^ ^]^ Using this material, a high EQE can be obtained in OLED devices, and light amplification can be realized via ASE in doped 4,4′‐bis(*N*‐carbazolyl)‐1,10‐biphenyl (*m*CBP) films. By increasing the doping concentration from 4 to 60 wt%, the ASE peak wavelength can be tuned from 740 to 799 nm with the threshold increasing from 4.7 to 36.7 µJ cm^−2^. Unlike other TADF materials with small Δ*E*_ST_s, compound 7 exhibited a relatively high Δ*E*_ST_ (as high as 0.37 eV). The authors suggested that nonadiabatic coupling effects between low‐lying excited states may facilitate the RISC process. This unusual mechanism endows the molecule with a high oscillator strength, which enables the achievement of a low‐threshold ASE. Based on compound **7**, a dimeric boron difluoride curcuminoid derivative (compound **8**) was designed to achieve an ASE of over 800 nm with a low threshold in doped thin films.^[^ ^49^ ^]^

In 2019, Fu et al. reported a new material (compound **6**, CAZ‐A) with a molecular structure similar to that of compound **7**.^[^ ^50^ ^]^ Lasing from 650 to 725 nm was achieved in the doped microrings. Very recently, the authors have also developed a set of difluoroboron‐based TADF gain materials (compounds **4a–4d**) with the boron atom directly connected to an oxygen atom and a nitrogen atom, with the exception of two fluorine atoms.^[^ ^46^, ^47^ ^]^ The lasing emission wavelengths of these materials can be modulated from green to yellow to red by changing the substitutes. In contrast to classical TADF materials, compounds **4a–4d** have large Δ*E*_ST_s (S_1_–T_1_) (0.74, 0.72, 0.56, and 0.57 eV respectively) which would hinder the RISC processes. However, the Δ*E*_ST_s (S_1_–T_2_) were calculated to be small enough (0.19, 0.02, 0.12, and 0.12 eV respectively) to permit the RISC processes to occur, and the large energy gaps between T_1_ and T_2_ make the transition of the internal conversion process difficult. Moreover, these compounds were calculated to have high spin−orbital coupling strengths between S_1_ and T_2_ compared with that between S_1_ and T_1_. According to these analyses, the RISC processes of compounds **4a–4d** were believed to be achieved between S_1_ state and T_2_ state. Therefore, in Table 1, the data of Δ*E*_ST_ (S_1_–T_2_) are provided instead of Δ*E*_ST_ (S_1_–T_1_) for these four compounds.

### 2.4 Design Principles of TADF Gain Materials

As only a few cases have been reported on TADF gain materials, the design principles of these materials need to be extracted from the existing reports. However, extracting general design principles from such a small number of cases is difficult. Therefore, only a few partial views are listed here based on existing reports and the design principles of TADF luminescent materials.

**Figure 3:**
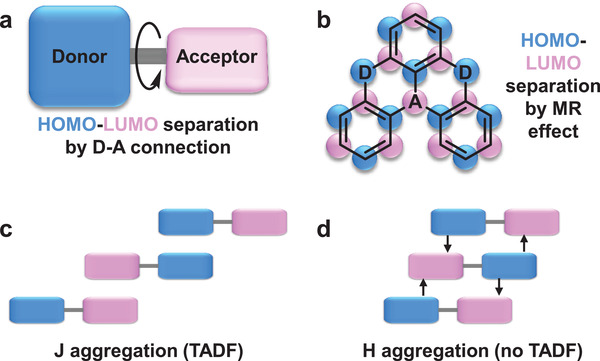
Design principle of a) conventional TADF materials and b) MR‐TADF materials. Schematics of c) J‐aggregation and d) H‐aggregation of compound **5**.

In traditional TADF materials, twisting the donor and acceptor moieties with large steric hindrance is important for achieving HOMO and LUMO separation to reduce Δ*E*_ST_ (**Figure** **3**a).^[^ ^10^, ^55^, ^56^ ^]^ In general, increasing the rigidity of the donor and acceptor skeletons helps in reducing the nonradiative losses caused by molecular vibration and thus improves the luminescence efficiency. However, for general organic gain materials, a four‐level system constructed using the molecular vibration level is an important prerequisite for realizing population inversion. Therefore, a reasonable limitation of molecular vibrations should be considered when designing TADF gain materials. For example, for compounds **2a** and **2b**, enhancing the rigidity of the molecular structures resulted in lower ASE thresholds.^[^ ^44^ ^]^ For the materials shown in Figure 2, the molecular rigidities are not too high, except for compounds **1a** and **1b**. Compounds **1a** and **1b** belong to another category of TADF material: multiresonant‐TADF (MR‐TADF) materials.^[^ ^58^, ^59^ ^]^ MR‐TADF materials are a class of fused polycyclic aromatic materials having mutually *ortho*‐disposed electron donating atom (donor) and electron deficient atom (acceptor) in a rigid planar polycyclic aromatic framework (Figure 3b).^[^ ^59^ ^]^ Due to the complementary resonance effects of the donor and acceptor the effective HOMO and LUMO separation and thus small Δ*E*_ST_ can be achieved. More importantly, the multiple resonance effect can cause a relatively high oscillator strength, and a high oscillator strength can lead to a large stimulated emission cross‐section, which is an important factor in achieving low‐threshold lasing.^[^ ^60^ ^]^ Hence, MR‐TADF materials may have unique advantages in achieving optical amplification. In addition to increasing molecular rigidity, the introduction of suitable substituents can also improve oscillator strength. In conclusion, appropriate molecular rigidity and substituents are important when designing TADF gain materials.

Molecular aggregation should be carefully considered in laser devices, particularly in non‐doped devices. As most TADF materials adopt a D‐A‐type molecular structure, intermolecular charge transfer (CT) interactions that can cause intermolecular orbital delocalization may affect the effective HOMO–LUMO separation in the aggregate state.^[^ ^61^ ^]^ For example, Fu et al. reported a gain material (compound **5**) with TADF properties in an aggregated state but without TADF properties in another aggregated state.^[^ ^51^ ^]^ As shown in Figure 3c,d, in J‐aggregation, the intermolecular CT interaction can be minimized, whereas in H‐aggregation, the intermolecular CT interaction can be enhanced. For J‐aggregated crystal, the ASE threshold was calculated to be 23.6 µJ cm^−2^ which is about three times lower than that of H‐aggregated crystal (93.2 µJ cm^−2^). It proved that the thresholds of TADF gain materials are lower than that of traditional fluorescence gain materials owing to the effective utilization of T_1_ excitons.

## 3 OSSLs Based on TADF Gain Materials

**Figure 4:**
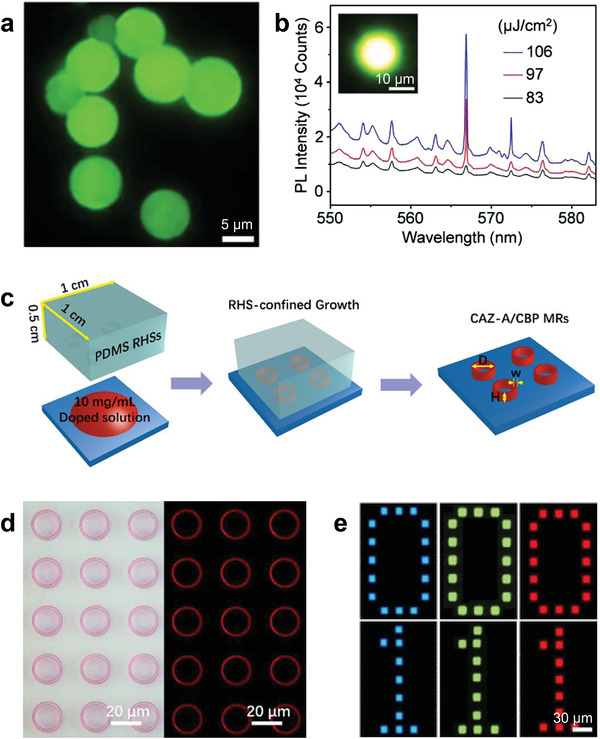
a) Fluorescence microscopy image of compound‐**3**‐doped PS microspheres. b) Laser spectrum of compound‐**3**‐doped PS microspheres. Inset: fluorescence microscopy image of a compound‐**3**‐doped PS microsphere above the lasing threshold. Reproduced with permission.^[^ ^45^ ^]^ Copyright 2020, Wiley‐VCH . c) Schematics of the poly‐(dimethylsiloxane) (PDMS) template‐confined solution‐growth method. d) Bright‐field (left) and fluorescence (right) microscopy images of compound‐**6**‐doped *m*CBP microring arrays. Reproduced with permission.^[^ ^50^ ^]^ Copyright 2019, American Chemical Society. e) Fluorescence microscopy images of *m*CBP microplates doped with different materials (compounds **1b**, **3**, and **7** from left to right) used as microlaser displays. Reproduced with permission.^[^ ^52^ ^]^ Copyright 2021, American Chemical Society.

Like any other laser, the OSSL consists of three basic units: pumping source, gain medium, and resonant cavity. Currently reported OSSLs are mainly operated under optically pumped conditions, and OSSLs under electrically pumped conditions are still difficult to achieve.^[^ ^26^ ^]^ Therefore, this section mainly discusses OSSLs based on the preparation of different types of resonators. Except for studies on the ASE properties of TADF gain materials without resonators, reports on OSSLs based on this type of material are limited. As summarized in **Figure** **4**, only a few studies have been conducted. It worth noted that all reported OSSLs are constructed by microcavities like microspheres and microcrystals of doped or pure gain materials. The microspheres and microcrystals with ultrasmooth surfaces and regular morphologies can work as gain materials and resonators simultaneously without extra reflective mirrors.

As mentioned in Section 2, Zhao et al. doped 4CzTPN (compound **3**) in PS and prepared PS microspheres with controllable and uniform sizes through solution self‐assembly using a surfactant (Figure 4a).^[^ ^45^ ^]^ These microspheres with perfect circular boundaries and ultrasmooth surfaces can be used as whispering‐gallery‐mode (WGM) resonators, in which photons can be strongly confined by means of successive total internal reflection along the sphere circumference. Under excitation by a pulsed laser, laser emission was achieved using these 4CzTPN‐doped microspheres (Figure 4b). The WGM‐type cavity resonance was proved by the linear relationship of *λ* ^2^/Δ*λ* versus diameters of the microspheres at *λ* = 562 nm, where Δ*λ* is the laser mode spacing. The cavity quality (*Q*) factor was calculated to be as high as 10^3^. PS microspheres are inexpensive and easy to prepare but are not suitable for electrically pumped lasers because of the nonconducting PS.

To achieve good electrical conductivity, organic semiconductive small‐molecular materials, such as *m*CBP, are usually used as host materials for active layers in OLEDs.^[^ ^62^, ^63^ ^]^ *m*CBP was used as a host material to study the ASE properties of organic gain materials by Adachi et al.^[^ ^48^, ^49^ ^]^ More importantly, *m*CBP microcrystals with specific morphologies can be easily fabricated using a poly‐(dimethylsiloxane) (PDMS) template‐confined solution‐growth method (Figure 4c).^[^ ^64^ ^]^ Microlasers can be constructed by doping gain molecules into *m*CBP microcrystals, which can then be used as resonators. In 2019, Fu et al. realized TADF lasers by doping CAZ‐A (compound **6**) into microring arrays with a high *Q* factor of ≈1300 (Figure 4d).^[^ ^50^ ^]^ Very recently, another study has been reported by Zhao et al. using the same strategy.^[^ ^52^ ^]^ By doping compounds **1a**, **3**, and **7**, respectively, into *m*CBP microplates using the aforementioned method, TADF microlasers with blue, green, and red emission colors were achieved. Vivid laser displays have also been demonstrated using microlaser arrays as display panels under programmable excitation (Figure 4e).

**Figure 5:**
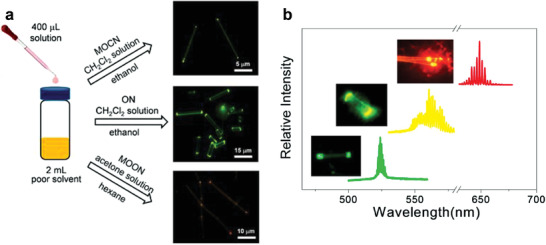
a) Schematics of the solution self‐assembly method and fluorescence microscopy images of microcrystals of compounds **4a–4c** (from top to bottom on the right side). b) Normalized lasing spectra of microcrystals of compounds **4a–4c** (from left to right). Inset: corresponding fluorescence microscopy images. Reproduced with permission.^[^ ^46^ ^]^ Copyright 2021, American Chemical Society.

Organic micro/nanocrystals with high purity, minimal defects, ordered structures, and potential applications in on‐chip devices have been widely used as active media and optical resonators simultaneously to develop organic lasers.^[^ ^65^, ^66^, ^67^ ^]^ Last year, Fu et al. reported four types of nondoped TADF microcrystals of compounds **4a–4d**.^[^ ^46^, ^47^ ^]^ One‐dimensional microcrystals that can function as Fabry–Pérot (FP) cavities can be formed via a simple solution self‐assembly method (**Figure** **5**a).^[^ ^66^, ^68^ ^]^ Lasing emissions with different colors were realized with low thresholds (Figure 5b). For the microrods of compound **4a**, the *Q* factor was estimated to be as high as ≈1313 at a wavelength of 525 nm. For the microribbons of compound **4d**, the *Q* factor was calculated to be as high as 2161. These studies provide an effective way to realize nondoped organic TADF laser diodes.

## 4 Challenges and Perspectives

With the continuous pursuit of electrically pumped OSSLs, TADF gain materials that can use T_1_ excitons via the RISC process have become a hot research topic. This mini‐review summarized the pioneering reports in recent years mainly from the viewpoint of materials. The types of molecular structures were introduced, and superficial design principles were extracted based on existing reports and the general design principles of TADF materials. OSSLs based on TADF gain materials with different types of resonators were also discussed. These studies provide good references for the realization of optical amplification using TADF materials.

However, certain aspects should be considered to realize electrically driven OSSLs with TADF gain materials. First, for most TADF materials, T_1_ excitons can only be effectively used in the RISC process in thermodynamics. In fact, in dynamics, the rate of RISC (*k*_RISC_) is very low compared with the radiative decay rate of S_1_ and the generation rate of T_1_ excitons under a high current density. For example, for compound **1b**, *k*_RISC_ is only on the order of 10^3^ s^−1^, which is four orders of magnitude lower than *k*_r_.^[^ ^43^ ^]^ The rate of intersystem crossing (*k*_ISC_) in TADF molecules is usually on the order of 10^6^–10^11^ s^−1^.^[^ ^10^ ^]^ In this case, most of the T_1_ excitons cannot be converted into S_1_ excitons in time. Therefore, the accumulation of T_1_ excitons cannot be completely avoided. Thus OLEDs based on TADF materials always face a serious roll‐off of the EQE. Second, the contradiction between the rapid RISC process and the high oscillator strength in molecular design may be another problem. To enhance the RISC rate, a twisted D‐A structure is usually adopted to reduce Δ*E*_ST_ through HOMO–LUMO separation. However, this type of structure always leads to a low oscillator strength. A part of the orbital overlap, instead of complete orthogonality, may help balance this contradiction. In addition, for new types of TADF materials such as MR‐TADF materials, this problem may be easier to overcome. Moreover, the poor photostability of TADF gain materials owing to thermal degradation at high current densities is another challenge. For electrically pumped OSSLs, the threshold is predicted to be very high. For example, the threshold of the first reported electrically pumped organic laser device is as high as 600 A cm^−2^.^[^ ^26^ ^]^ On the one hand, to improve the photostability, the structural stability of TADF gain materials should be fully considered in molecular design; on the other hand, a lower laser threshold will be more helpful by reducing the pumping energy considering the nature of organic materials. In theory, for TADF gain materials, the lasing threshold can be reduced dramatically because that most of energy losses caused by triplet excitons in traditional fluorescent gain materials can be avoided. However, the progress of TADF gain materials is still in the early stage and the lasing thresholds of reported TADF materials are not lower than that of fluorescent gain materials. Nevertheless, the TADF gain material still has a huge potential. We believe that with the development of TADF gain materials the lasing threshold can be lowered greatly.

As mentioned above, to realize electrically driven OSSLs some decisive factors should be considered when designing TADF gain materials in addition to previously reported design principles of TADF luminescent materials: (1) High *k*_RISC_ and short TADF lifetime. The faster the RISC process, the more T_1_ excitons that can be recycled. (2) Shot lifetime of prompt fluorescence; The fluorescence lifetime should be as short as several nanoseconds to avoid the nonradiative decay of singlet excitons. In fact, the lifetimes of most effective gain molecules were shorter than 1 ns. (3) High oscillator strength; Theorical calculations of oscillator strength can be employed to predict the lasing properties of designed molecules before tedious synthetic work. (4) Robust molecular structure; The thermal degradation of organic gain materials at high current densities is still one of the most important problems compared with that of inorganic materials. For conventional TADF materials, introducing other photochemical processes, such as ESIPT, may be helpful in improving their laser activities because a more effective four‐energy‐level system can be easily formed for ESIPT materials. This will result in easier population inversion and thus benefit stimulated emissions.^[^ ^24^ ^]^

In addition to TADF materials, other kinds of materials can utilize T_1_ excitons, such as hybridized local and charge‐transfer and triplet–triplet annihilation upconversion (TTA‐UC) materials.^[^ ^69^, ^70^ ^]^ Studies using the TTA‐UC process to recycle T_1_ excitons have been reported, and quasi‐continuous‐wave organic lasers have been achieved.^[^ ^42^ ^]^ Although the theoretical efficiency of TTA‐UC materials (a maximum of 62.5%) is lower than that of TADF materials (a maximum of 100%), the TTA process can be very fast at high concentrations of T_1_ excitons.^[^ ^71^ ^]^ Therefore, the utilization efficiency of triplet excitons may be higher for TTA‐UC materials. Hence OLED devices based on TTA‐UC are more stable.^[^ ^72^ ^]^ However, TTA‐UC materials with laser activity have not yet been reported.

In summary, considerable work remains to be done to achieve electrically pumped OSSLs based on TADF gain materials. The most important problem is the lack of effective TADF gain materials. Only when more TADF gain materials are designed and synthesized can the structure–property relationship be studied. A greater choice of materials will provide more opportunities to overcome the existing problems in realizing practical current‐injected OSSLs. In addition to the material design, carefully designed resonators may help achieve a lower threshold. For example, in the first reported electrically pumped OSSL, a mixed‐order distributed feedback (DFB) structure was used.^[^ ^26^ ^]^ The DFB and distributed Bragg reflector (DBR) structures have been demonstrated to be more effective resonators.^[^ ^73^, ^74^, ^75^, ^76^ ^]^ Therefore, the use of DFB and DBR structures may accelerate the development of TADF lasers. We believe that our review will attract more scientists to this field, aiding the advances in TADF gain materials, and facilitate the development of electrically driven OSSLs.

## Conflict of Interest

The authors declare no conflict of interest.
